# Serum IL-18 is a specific biomarker for Macrophage Activation Syndrome across several autoinflammatory diseases

**DOI:** 10.1186/1546-0096-13-S1-P10

**Published:** 2015-09-28

**Authors:** SW Canna, AA de Jesus, G Shi, Y Huang, GA Montealegre Sanchez, I Gery, R Goldbach-Mansky

**Affiliations:** 1 NIAMS/NIH, Bethesda, USA; 2 NEI/NIH, Bethesda, USA

## Question

IL-18 is a pro-inflammatory cytokine produced by a variety of myeloid and non-hematopoietic cells. It is canonically associated with enhancing interferon gamma (IFNg) and cytotoxicity in collaboration with IL-12p70, IL-15, or type I IFN. However, in other contexts IL-18 can promote IL-17, IL-22, or allergic responses. Macrophage Activation Syndrome (MAS) is a sepsis-like syndrome that has been associated with elevated serum IL-18 in systemic Juvenile Idiopathic Arthritis, Stills disease, and XIAP-deficiency. We sought to characterize IL-18 and associated cytokines in a cohort of patients with a variety of monogenic or complex autoinflammatory syndromes.

## Methods

Serum IL-18 was measured across several platforms and normalized to healthy controls run in the same batch. For many patients, IL-18 binding protein (IL-18BP) and IL-37 were measured from the same sample. Results were correlated with clinical laboratory findings, most notably acute phase reactants like C-reactive protein and erythrocyte sedimentation rate obtained on the same date.

## Results

We found three patterns of serum IL-18: 1) normal IL-18 in healthy controls, patients with STING mutations, patients with chronic non-bacterial osteomyelitis (CNO), and patients with deficiency of IL-1 receptor antagonist (DIRA); 2) Mild elevation (less than 10-fold above normal) of serum IL-18 in patients with defects in NLRP3 (Cyropyrin Associated Periodic Syndromes, CAPS) or proteasomal defects (Chronic Atypical Neutrophilic Dermatosis Lipodystrophy Elevated Temperature, CANDLE); and 3) extraordinary elevation (100 to 500 fold above normal) in patients with a history of MAS regardless of disease activity. Multiple serial IL-18 measurements were made in a patient harboring an *NLRC4* mutation, as well as a patient with clinical NOMID (including severe epiphyseal overgrowth) with no detectable germ-line or somatic gene defect who had multiple severe MAS episodes. There are two endogenous antagonists of IL-18: IL-18BP and IL-37. These cytokines correlated moderately with CRP, but not with serum IL-18.

## Conclusions

Our data suggest that extreme elevation of serum IL-18, particularly in the absence of acute inflammation, is a unique biomarker for MAS risk across many autoinflammatory phenotypes. The mechanisms by which chronic elevation of IL-18 may promote the MAS phenotype need to be further investigated.

